# Anakinra: efficacy in the management of fever during neutropenia and mucositis in autologous stem cell transplantation (AFFECT-2)—study protocol for a multicenter randomized double-blind placebo-controlled trial

**DOI:** 10.1186/s13063-020-04847-5

**Published:** 2020-11-23

**Authors:** Charlotte E. M. de Mooij, Lenneke F. J. van Groningen, Anton F. J. de Haan, Bart J. Biemond, Martijn Bakker, Walter J. F. M. van der Velden, Nicole M. A. Blijlevens

**Affiliations:** 1 Radboud Institute of Health Sciences, Department of Hematology, Radboud University Medical Center, PO Box 9101, 6500 HB Nijmegen, the Netherlands; 2 Department for Health Evidence, Radboud University Medical Center, Nijmegen, the Netherlands; 3 Department of Hematology, Amsterdam UMC, Amsterdam, the Netherlands; 4 Department of Hematology, University Medical Center Groningen, Groningen, the Netherlands

**Keywords:** Febrile neutropenia, Mucositis, Hematopoietic stem cell transplantation, Interleukin-1, Anakinra, Protocol, Randomized controlled trial, Multiple myeloma, Microbiota

## Abstract

**Background:**

Since decades, fever and infections have been the most important complications of intensive chemotherapy and hematopoietic stem cell transplantation (HSCT) in the treatment of hematologic malignancies. Neutropenia has long been considered to be the most important risk factor for these complications. However, recent studies have shown that not neutropenia, but the development of mucositis is the most important cause of these complications. Currently, limited options for the prevention and treatment of mucositis are available, of which most are only supportive. The pro-inflammatory cytokine interleukin-1 (IL-1) plays a crucial role in the pathogenesis of mucositis. Pre-clinical studies of chemotherapy-induced mucositis have shown that recombinant human IL-1 receptor antagonist anakinra significantly ameliorated intestinal mucositis. In our pilot study AFFECT-1, we examined the safety and maximal tolerated dose of anakinra in patients with multiple myeloma, treated with high-dose melphalan (HDM) and autologous HSCT, selecting a dose of 300 mg daily for the phase IIb trial. The aim of the AFFECT-2 study is to determine the efficacy of anakinra in preventing fever during neutropenia (FN) and mucositis in this study population.

**Methods/design:**

A multicenter, randomized, placebo-controlled, double-blind phase IIb trial will be conducted. Ninety patients with multiple myeloma scheduled for treatment with HDM and autologous HSCT will be included. Patients will be randomized between intravenous treatment with anakinra (300 mg) or placebo. Each group will be treated from day − 2 (day of HDM; day 0 is HSCT) up until day + 12. Outcome measures will be assessed at baseline, during admission, at discharge or day + 30, at day + 90, and + 1 year. The primary outcome will be reduction of FN. Secondary outcome measures include mucositis scores, bloodstream infections, citrulline levels, quality of life, and fatigue severity.

**Discussion:**

The AFFECT-2 trial will examine the efficacy of anakinra in the management of fever during neutropenia and mucositis in patients with multiple myeloma treated with HDM and autologous HSCT. The results of this study may provide a new treatment option for these important complications. Also, this study will give us more insight in the pathophysiology of mucositis, including the role of IL-1 and the role of the microbiota in mucositis.

**Trial registration:**

Clinicaltrials.gov NCT04099901. Registered on September 23, 2019. EudraCT: 2018-005046-10.

## Administrative information

**Table:** 

Title {1}	Anakinra: Efficacy in the Management of Fever During Neutropenia and Mucositis in Autologous Stem Cell Transplantation (AFFECT-2) – Study Protocol for a Multi-center Randomized Double-blind Placebo-controlled Trial
Trial registration {2a and 2b}	Clinicaltrials.gov: NCT04099901 Clinicaltrials.gov registration date: September 23th 2019. EudraCT: 2018-005046-10
Protocol version {3}	Protocol version 1.1, 29-05-2019
Funding {4}	Dutch Cancer Society (DCS; Dutch: KWF Kankerbestrijding), project number 11236 / 2017–2
Author details {5a}	^1^Radboud Institute of Health Sciences, Department of Hematology, Radboud University Medical Center, Nijmegen, the Netherlands ^2^Department for Health Evidence, Radboud University Medical Center, Nijmegen, the Netherlands ^3^Department of Hematology, Amsterdam UMC, Amsterdam; ^4^Department of Hematology, University Medical Center Groningen, Groningen;
Name and contact information for the trial sponsor {5b}	Radboud University Medical Center PO box 91016500 HB, Nijmegen, The Netherlands.Website: https://www.radboudumc.nl/en/research
Role of sponsor {5c}	The sponsor and funding body have no role in the study design, data collection, data analysis, data interpretation, writing the report, or decision to submit the report for publication.

## Introduction

### Background and rationale {6a}

Most hematologic malignancies require treatment with chemotherapy and radiotherapy. Increasing the intensity of these therapy regimens has resulted in better disease control with lower relapse rates and increased progression-free and overall survival. However, the beneficial effects are still offset by significant treatment-related morbidity and mortality resulting from toxic side effects, including neutropenia, mucositis, and infections. Consequently, patients with comorbidities, especially older individuals, often do not receive these therapies and cannot benefit from intensive chemotherapy. Fever during neutropenia (FN) and mucositis are the most common causes for the delay of treatment, reductions of dosage, and even permanent cessation of anticancer therapy, which results in a significantly reduced chance of cure. Moreover, the extensive use of antimicrobial agents in these patients has been accompanied by certain risks, including the development of antimicrobial resistance, the distortion of the healthy gut microbiota, and increased costs.

#### Febrile mucositis

Although it is generally assumed that FN is related to infection, that assumption has not always been proven to be the case. For instance, despite the use of antimicrobial agents, 70–90% of patients receiving intensive chemotherapy ultimately develop FN [1]. Moreover, fever remains unexplained in 30–40% of neutropenic patients, since clinically or microbiologically, no focus of the infection can be found [2].

Several studies have shown that mucositis or mucosal damage of the intestinal tract and oral cavity (mucosal barrier injury, MBI) is an independent risk factor for the occurrence of infection, mainly bloodstream infections, FN, and inflammatory complications [2–4]. Moreover, the median time of onset of FN is at day 12 after starting chemotherapy, when mucositis is at its worst [5].

Cytotoxic therapy induces mucosal barrier breach, which leads to translocation of micro-organisms. Furthermore, tissue damage is followed by activation of host response mechanisms, with release of cytokines and chemokines. This leads to a strong inflammatory response that manifests itself primarily with fever, even in the absence of infection.

The amino acid citrulline, which reflects enterocyte mass, has shown to be an objective, reproducible, and reliable biomarker to determine the degree of intestinal mucositis [6, 7]. Decreased citrulline levels, but not neutrophil count, have been shown to correlate with systemic inflammation and fever, even in the absence of infection. Therefore, the term febrile neutropenia does not suffice and should be at least supplemented with the concept of “febrile mucositis,” which better reflects the pathophysiological background [8]. According to this concept, reducing mucosal damage may also reduce the incidence of fever and infections. In a study by Spielberger, the use of recombinant keratinocyte growth factor (palifermin) significantly decreased the incidence of oral mucositis in patients with an HSCT after conditioning with radiotherapy-containing regimen. The incidence of fever during neutropenia in this study also significantly decreased, with 20%, despite the fact that the drug had no effect on the duration of neutropenia [9].

#### Unmet need for treatment of mucositis

Clinically, mucositis causes pain, diarrhea, and an increase in the use of total parenteral nutrition and pain medication. Also, it is associated with a decreased quality of life, dose reductions, and interruptions or even cessation of chemotherapy, as well as an increase in healthcare costs, prolonged hospital stays, and mortality [2, 3, 8, 10, 11].

Moreover, increasing data show that disturbance of the intestinal microbiota (dysbiosis), which for an important part is caused by the extensive use of antibiotics, is associated with the development of intestinal mucositis and even decreased survival [12–14].

Currently, only limited strategies are available for the prevention or treatment of mucositis. Although palifermin has shown to ameliorate oral mucositis [9], it proved to be context dependent, so not widely applicable. Moreover, the drug failed to significantly reduce intestinal mucositis [15, 16].

An example of a treatment that is associated with a high incidence of mucositis is high-dose melphalan. This treatment, followed by autologous HSCT is currently considered the standard treatment of patients with multiple myeloma younger than 65 years and fit patients in good clinical condition. Importantly, recent studies have confirmed that this should remain the standard of care [17–20]. High-dose melphalan (HDM) induces considerable oral and intestinal mucositis and comes with an incidence of FN of approximately 80–85% [2, 11].

#### Targeting IL-1 in chemotherapy-induced mucositis

Interleukin-1 (IL-1α and IL-1β) is an important cytokine, involved in many physiological processes and diseases. The most important function of IL-1, which binds to the IL-1 receptor, is regulating local and systemic inflammatory processes that contribute to the protective immune response against infections. Dysregulated production and signaling of these cytokines aggravates tissue damage during infections and inflammatory diseases. Safe IL-1 inhibitors are currently available, such as the IL-1 receptor antagonist (IL-1RA) anakinra, and the anti-IL-1β antibody canakinumab. In the past few years, IL-1 was shown to play a crucial role in the initiation and propagation of mucositis [21]. In murine models of chemotherapy-induced mucositis, the role of IL-1, as well as the effect of IL-1 inhibition using IL-1RA and anti-IL-1β antibodies, was studied in a number of chemotherapeutical regimens. It was demonstrated that IL-1β is responsible for the increased permeability of the mucosal barrier that occurs in an early phase of mucositis, due to dysregulation of epithelial tight junctions [22, 23]. Subsequently, this increased intestinal permeability facilitates translocation of microorganisms and microbe-associated molecular patterns (MAMPs), which further induces inflammation [22].

The increased expression of IL-1β follows a similar course over time as epithelial cell apoptosis. Treatment with IL-1RA resulted in reduced crypt cell apoptosis, histologically attenuated intestinal damage with preservation of crypts and villi, and clinically, a decrease in diarrhea and weight loss [22, 24–27]. Moreover, no detrimental effects were seen on anti-tumor effects of chemotherapeutics, and survival in animals treated with IL-1 inhibition improved [24–26]. Based on these data, IL-1 appears to be a promising target for the prevention and treatment of mucositis and resulting inflammatory complications.

#### AFFECT-1 study

From March 2018 to April 2019, we conducted the AFFECT-1 study (Safety and efficacy of interleukin-1 inhibitor anakinra for the amelioration of fever during neutropenia and mucositis in patients with multiple myeloma receiving an autologous hematopoietic stem cell transplantation after high-dose melphalan, ClinicalTrials.gov identifier NCT03233776). The main goal of this phase IIa study was to determine the safety of anakinra in this study population. Using a 3 + 3 design, 3 dosages of anakinra were tested in order to determine the maximum tolerated dose (100, 200, or 300 mg) in terms of safety, based on dose-limiting toxicities.

A total of nine patients have been treated with anakinra; three per dose cohort. Patients did not complain of side effects that could be linked to the use of anakinra. No adverse events occurred, other than adverse events that are usually seen in the treatment with high-dose melphalan (e.g., nausea, diarrhea, cytopenias). Therefore, we determined that the maximum tolerated dose of 300 mg anakinra could be used in the AFFECT-2 study.

#### Rationale

The concept of “febrile mucositis” implicates that new strategies that aim at prevention and treatment of mucositis will also reduce the incidence of FN and related complications and consequently the use of antimicrobial agents and other resources. We hypothesize that inhibition of the IL-1 pathway, in patients receiving intensive chemotherapy in the context of HSCT, can decrease the incidence of intestinal mucositis and consequently fever during neutropenia. Moreover, we expect that by using anakinra, thereby avoiding the use of broad-spectrum antimicrobial agents, the gut microbiota will be preserved.

### Objectives {7}

The primary objective of this study is to evaluate the efficacy of anakinra in the prevention of fever during neutropenia in patients receiving HDM in the preparation for an autologous HSCT. Secondary objectives are to describe the effect of anakinra on the incidence and severity of mucositis. Secondary objectives focused on translational research in this population are to describe the composition of and changes in the gut microbiota of patients receiving an autologous HSCT, to evaluate the role of the gut microbiota in the pathogenesis of mucositis and to collect data for validation of potential biomarkers reflecting the severity of intestinal mucositis.

### Trial design {8}

The AFFECT-2 trial is designed as a multicenter, parallel group, superiority, randomized, placebo-controlled, double-blind phase IIb trial comparing the efficacy of once daily intravenous anakinra against placebo in patients with multiple myeloma treated with HDM and autologous HSCT, to reduce the incidence of fever during neutropenia. Subjects will be stratified by investigational site. Randomization will be performed as block randomization with a 1:1 allocation.

## Methods: participants, interventions, and outcomes

### Study setting {9}

**Fig. 1:**
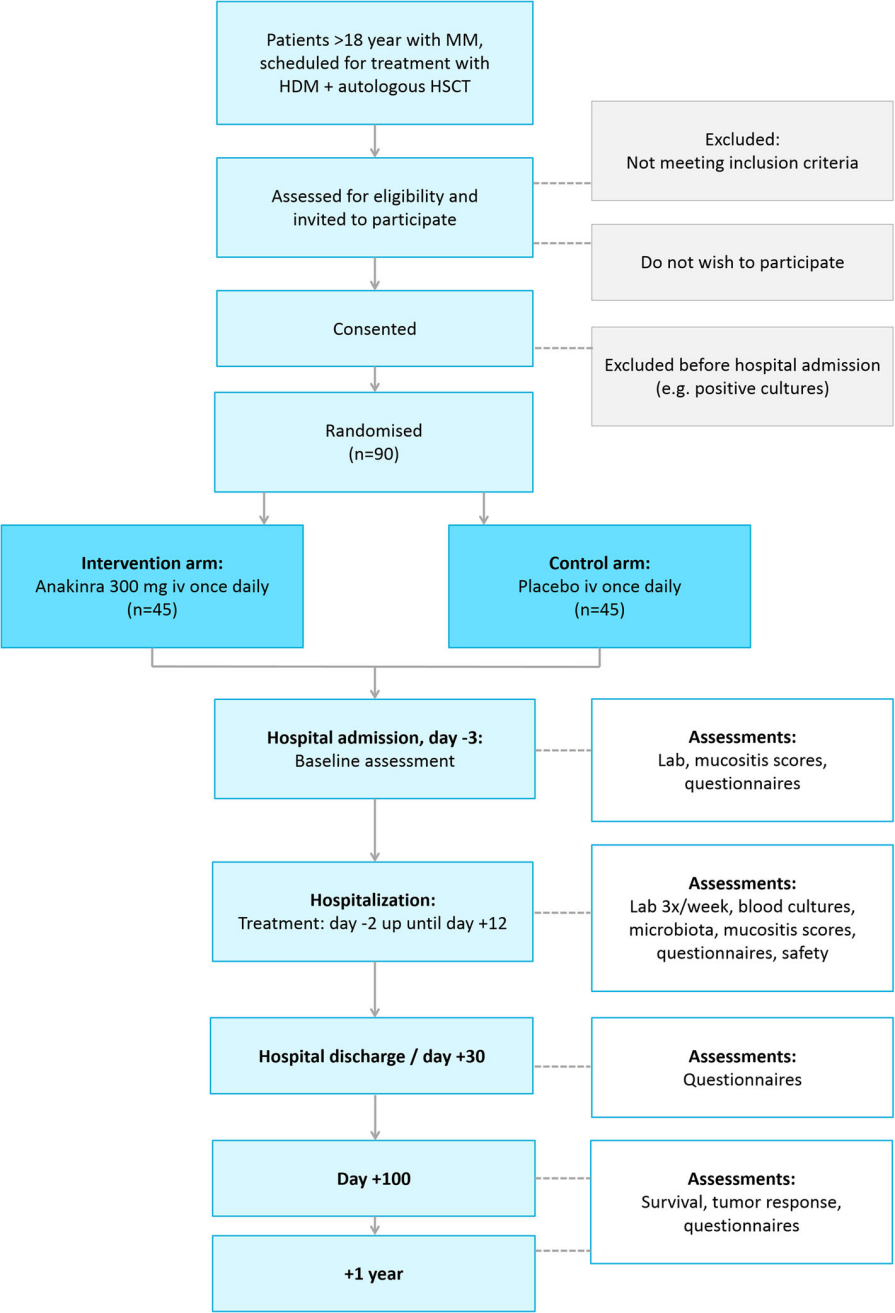
Study flowchart

The study will be performed at the departments of hematology in the Radboud University Medical Center Nijmegen (coordinating site), the Amsterdam UMC and the University Medical Center Groningen (UMCG), which are all academic, tertiary referral hospitals located in the Netherlands. All patients of 18 years and older planned for non-ambulant treatment with HDM and autologous HSCT will be considered for participation (Fig. 1).

### Eligibility criteria {10}

#### Inclusion criteria

In order to be eligible for participation, patients must meet all of the following inclusion criteria:

- Aged ≥ 18 years
- Diagnosed with multiple myeloma
- Scheduled to receive an autologous HSCT after myeloablative therapy with HDM
- Managed with a central venous catheter (triple- or quadruple lumen)
- Able and willing to participate
- Provided written informed consent
- Negative serology for active hepatitis B and C
- Negative serology for HIV
- No known hypersensitivity to *Escherichia coli*-derived products or any components of anakinra
- Women of childbearing potential and men must agree to use adequate contraception (hormonal or barrier method of birth control; abstinence) prior to study entry, for the duration of study participation (during treatment with study medication), and for 30 days after the last dose.

#### Exclusion criteria

A potential participant who meets any of the following criteria will be excluded from participation in this study:

- Inability to understand the nature and extent of the trial and the procedures required
- Enrollment in any other investigational treatment study or use of an investigational agent during the stem cell transplantation (this means studies in multiple myeloma regarding induction or maintenance treatment are permitted)
- Women who are pregnant or nursing
- Diagnosed with amyloidosis or light-chain deposition disease
- ALT or AST greater than 2.0× upper limit of normal (ULN) of the local laboratory values
- Bilirubin levels greater than 2.0× upper limit of normal (ULN) of the local laboratory values, except for benign non-malignant indirect hyperbilirubinemia such as Gilbert syndrome
- Impaired renal function with eGFR < 40 ml/min
- Received a live vaccine during the 3 months prior to baseline visit
- Recent use of IL-1 antagonist, such as anakinra, rilonacept, or canakinumab, within 3 months prior to baseline visit
- Treatment with TNFα-inhibiting agents (such as etanercept, adalimumab, infliximab, certolizumab, and golimumab)
- Uncontrolled bacterial or viral infections, or fungal infections, at the start of therapy
- Colonization with methicillin-resistant *Staphylococcus aureus* (MRSA), carbapenemase-producing Enterobacteriaceae (CPE), or vancomycin-resistant enterococci (VRE) prior to registration
- Gram-negative colonization resistant to prophylaxis with ciprofloxacin or colistin/cotrimoxazole
- Subjects who are not able to receive antibacterial prophylaxis with ciprofloxacin or colistin/cotrimoxazole (because of hypersensitivity or drug interactions)
- Subjects with an active solid malignancy prior to registration, with the exception of cutaneous basal or squamous cell carcinomas
- History of mycobacterial infection
- Subjects with intrinsic disorders of the gastrointestinal (GI) tract, including, but not limited to, Crohn’s disease, ulcerative colitis, celiac disease, and short bowel syndrome
- Subject has any concurrent medical or psychiatric condition or disease that is likely to interfere with the study procedures or results or that, in the opinion of the investigator, would constitute a hazard for participating in this study.

#### Specific exclusion criterium for potential study participants in Amsterdam UMC

Patients who will be treated in ambulatory care after HSCT (early discharge after transplant) cannot be included in the study for logistic reasons (i.e., daily infusion with anakinra from day − 2 until day + 12, daily mucositis scores).

#### Screening procedures

To evaluate eligibility, available information obtained through standard care will be used, including medical history, laboratory analysis, and microbiological research (including viral serology). On indication (in patients with risk factors for latent infection), a Quantiferon test will be performed to rule out latent tuberculosis. Oral and rectal swabs to rule out colonization with MRSA, CPE, VRE, or colonization with Gram-negative bacteria resistant to prophylaxis with ciprofloxacin or colistin/cotrimoxazole will be performed. Except for colonization cultures, all tests are routinely performed as a part of standard pre-transplantation evaluation in these patients.

### Who will take informed consent? {26a}

Potential participants receive oral information by researchers trained in Good Clinical Practice (GCP), as well as written information. After time for consideration, written informed consent is taken from patients willing to participate in the trial.

### Additional consent provisions for collection and use of participant data and biological specimens {26b}

#### Participants treated in the Radboud University Medical Center

All participants in the Radboudumc are asked at an earlier time point (unrelated to the AFFECT-2 study) if they will participate in the Biobank Hematology (HEMBB, IRB Registration number: CMO Arnhem-Nijmegen 2013/064), in which remaining material will be stored and potentially used for future translational research, for the development and evaluation of treatment for hematological cancers, as well as for supportive care during treatment.

### Interventions

#### Explanation for the choice of comparators {6b}

The comparator is placebo, which is chosen to enable a double-blind study setting, since no other treatment options are available. Patients receive treatment with anakinra/placebo on top of standard of care.

### Intervention description {11a}

#### Routine care

All participants will be managed with a central venous catheter (CVC) and will be accommodated in a (HEPA-filtered) room equipped with laminar flow. Participants will be admitted on day − 3 and will receive conditioning with HDM (200 mg/m2) on day − 2 and autologous stem cell infusion (minimal CD34+ SC dose 2.5 × 10^6^/kg).) on day 0. Participants will receive—according to international guidelines (IDSA, ECIL)—the fluoroquinolone ciprofloxacin (only oral) for prophylaxis against bacteremia due to gram-negative bacilli, as well as fluconazole (oral or iv) as *Candida* prophylaxis on indication, and (val)acyclovir (oral or iv) as viral prophylaxis in case of herpes seropositivity. In case of gram-negative colonization resistant to ciprofloxacin, cotrimoxazole/colistin (oral) can be used as prophylaxis.

In case of fever, defined as a tympanic temperature ≥ 38.5 °C, empirical therapy with ceftazidime shall be initiated.

#### Study treatment

The intervention will be administration of a daily intravenous dose of 300 mg anakinra, starting on day − 2, until day + 12. The comparator will be administration of a daily intravenous dose of placebo, starting on day − 2, until day + 12. Anakinra/placebo will be given in a volume of 50 mL, administered with an infusion speed of 30 ml/h.

#### Anakinra

Anakinra (Kineret®, Swedish Orphan Biovitrum AB) is a recombinant non-glycosylated form of the human interleukin-1 receptor antagonist. Besides the inclusion of a single methionine residue at its amino terminus, it is identical to the naturally present human IL-1 receptor antagonist. Anakinra is produced by recombinant DNA technology in *Escherichia coli* cells. It neutralizes the biological activity of interleukin-1α (IL-1α) and interleukin-1β (IL-1β) by competitively binding to the interleukin-1 receptor type I (IL-1RI). Its binding does not induce signaling or cell activation.

Currently, Kineret® is registered for treatment of adults with rheumatoid arthritis (RA) and adults, adolescents, children, and infants for the treatment of cryopyrin-associated periodic syndromes (CAPS).

Regarding study treatment, pre-packed solutions of subcutaneous anakinra will be added to a 50-mL syringe and will be diluted with sterile NaCl 0.9% to a volume of 50 mL. Syringes will be kept at 2 to 8 °C.

#### Placebo

As placebo, sodium chloride will be used, since anakinra is also transparent and has no specific characteristics. A volume of 50 mL sterile NaCl 0.9% will be added to a 50-mL syringe. Syringes will be kept at 2 to 8 °C.

### Criteria for discontinuing or modifying allocated interventions {11b}

#### Investigational treatment discontinuation

Investigational treatment is discontinued when any of the following occurs:

- Patient refusal or non-compliance
- Unacceptable or life-threatening toxicity
- Adverse event(s) that, in the judgment of the investigator, may cause severe or permanent harm or which rule out continuation of the study
- Withdrawal of consent
- Persistent bacteremia
- Occurrence of a suspected unexpected serious adverse event (SUSAR)

#### Dosage modifications

During the study, dosage modifications are not allowed. If subjects develop renal impairment with an eGFR < 30 ml/min, dosage will not be adjusted, but study medication will be stopped.

#### Specific criteria for withdrawal from the study

Subjects may be withdrawn from the study treatment and assessments at any time for any of the following reasons:

- Withdrawal of informed consent.
- Non-compliance with the protocol.
- Use of any concomitant medication which according to the judgment of the investigator may interfere the objectives of the study.
- For safety reasons
- Medical opinion dictates withdrawal in the interest of the patient

#### Replacement of individual subjects after withdrawal

If subjects are non-evaluable for the study objectives after withdrawal, we will consider including additional subjects according to our intention to evaluate 90 subjects.

In principle, inclusion will continue until 100 subjects are included, to assure a number of 90 evaluable subjects as estimated to be necessary. However, if 90 evaluable subjects already have completed the study treatment phase (initial hospital admission), inclusion will stop at that time point.

### Strategies to improve adherence to interventions {11c}

Research nurses check during the treatment period whether intervention protocols are adhered to and give feedback when this is not the case. No procedures are taken to ensure patient adherence, since patients are admitted during the treatment period and receive study medication intravenously.

### Relevant concomitant care permitted or prohibited during the trial {11d}

#### Medication or therapies prohibited during the study

These therapies are prohibited during the treatment phase of the study (hospital admission for HDM and HSCT):

- Drugs or therapies for the prevention or treatment of mucositis (oral or intestinal). Examples include palifermin (Kepivance®, human recombinant keratinocyte growth factor), loperamide, and octreotide.
- The use of hematopoietic growth factors such as (peg)filgastrim
- Antiphlogistic therapy such as acetaminophen and non-steroidal anti-inflammatory drugs (NSAIDs). One exception: a single dose of acetaminophen is allowed prior to the stem cell (SC) infusion (standard SC infusion protocol).
- The use of gram-positive prophylaxis (oral or iv)
- Glucocorticoids are allowed during the study in three situations: first, a single dose of prednisolone is allowed prior to the SC infusion (standard SC infusion protocol); second, three doses of dexamethasone on day − 2, − 1, and 0 as antiemetic (standard protocol); and third, in case of alpha-streptococcal syndrome (acute respiratory distress syndrome (ARDS), severe sepsis, or septic shock)

Furthermore, administration of live vaccines will not be allowed throughout the course of the whole study.

#### Allowed medication

Aside from the abovementioned drugs, subjects are allowed to use all the drugs which are prescribed by their treating hematologists.

### Provisions for post-trial care {30}

#### Post-trial care

Standard care is provided after participants have finished the study treatment phase (initial hospital admission).

#### Compensation for injury

An insurance has been taken out for the participants in this clinical trial. This insurance covers losses caused by death or injury resulting from participation in the clinical trial, which reveals itself during the participation of the subject in the clinical trial or within 4 years thereafter.

### Outcomes {12}

#### Primary endpoint

The primary study endpoint will be the reduction of the incidence of fever during neutropenia. This is defined as a tympanic temperature ≥ 38.5 °C and an absolute neutrophil count (ANC) < 0.5 × 10^9^/l, or expected to fall below 0.5 × 10^9^/l in the next 48 h. All episodes of fever occurring due to blood and blood product transfusions will be excluded.

#### Secondary endpoints

Secondary endpoints will be:

- Reduction in incidence of mucositis-related fever
- Maximum CRP level on day + 9–10
- Daily mean CRP level
- Intestinal mucositis as measured by (the area-under-the-curve of) citrulline levels
- Clinical mucositis as determined by the daily mouth and gut scores
- Days with fever (≥ 38.2 °C)
- Days with fever (≥ 38.5 °C)
- Mean daily morning temperature
- Area under the curve of daily morning temperature
- Incidence of bloodstream infections, i.e., bacteremia
- Type of bloodstream infections
- Incidence of persistently positive blood cultures on day + 4
- Quality of life according to the EORTC QLQ-C30
- Severity of fatigue as the score measured by the validated FACIT-Fatigue scale
- Tumor response evaluation (100 days and 1 year)
- Short-term overall survival (100 days and 1 year)
- Length of hospital stay in days
- Use of systemic antimicrobial agents (incidence and duration)
- Use of analgesic drugs (incidence and duration)
- Use of total parenteral nutrition (TPN) (incidence and duration)

It should be noted that bacteremia is defined by any single blood culture-yielding bacteria except for coagulase-negative staphylococci, which require the same strain to be recovered from two separate cultures. This also applies to “persistently positive blood cultures.”

### Participant timeline {13}

See Fig. 2.

**Fig. 2:**
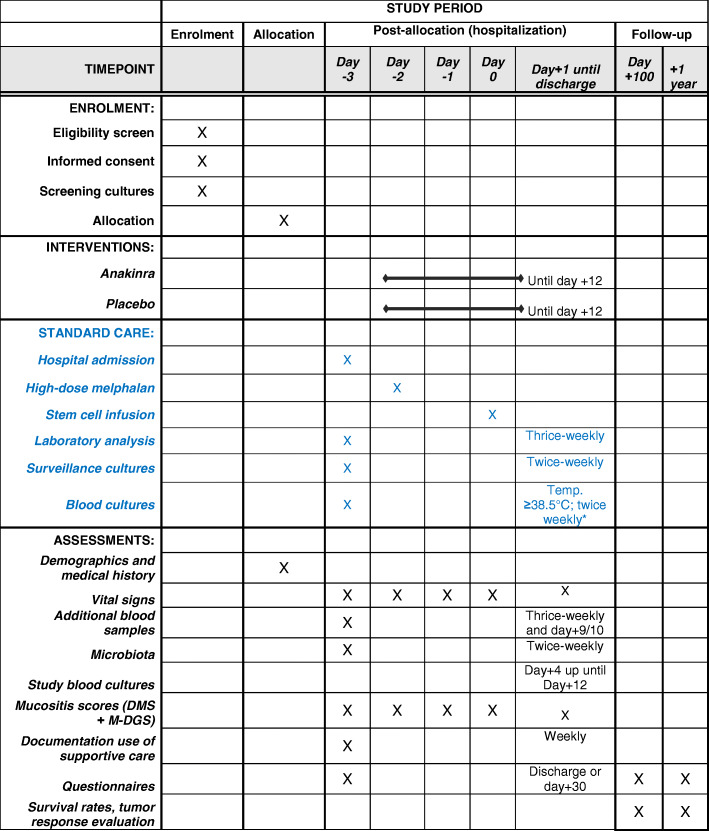
Overview of study procedures and assessments

### Sample size {14}

Our assumption is that that the rate of fever during neutropenia will be 80% in the placebo group and 50% in the intervention group.

A number of 45 subjects is required per study arm to detect a 30% reduction (from 80 to 50%) in the incidence of fever during neutropenia, with a two-sided test, an *α* of 0.05 and a power of 80%.

At the departments of hematology of the participating centers, a minimum of 40 autologous stem cell transplantations are performed for multiple myeloma each year.

A total of 90 subjects will be included. With an expected accrual rate of 15 subjects per year per center, entry will be completed in about 2 years.

### Recruitment {15}

The study team at each study center will be responsible for identifying potential participants. A study team member will give both oral and written information to potential participants. If eligible and willing to take part, potential participants will be asked to give written informed consent.

### Assignment of interventions: allocation

#### Sequence generation {16a}

Participants who provide written informed consent and fulfill the eligibility criteria will be randomly allocated to either the intervention arm (anakinra) or control arm (placebo) with a 1:1 ratio using computer randomization. Randomization will be stratified by study site. Variable block randomization will be used, with block sizes of 2, 4, and 6. The randomization procedure with treatment allocation will be done by an authorized study team member, using Castor Electronic Data Capture (EDC), a web-based system that also will be used for data entry.

#### Concealment mechanism {16b}

The web-based randomization system ensures allocation concealment.

#### Implementation {16c}

The allocation sequence generation is imbedded in Castor EDC. Study team members at the Clinical Trials Unit of every study site will enroll participants and randomize them using the web-based randomization system.

### Assignment of interventions: blinding

#### Who will be blinded {17a}

Study team members, healthcare providers, and participants will be blinded to both the randomization schedule and subsequent treatment allocation. The sequence will be concealed at all times throughout the study, except for the hospital trial pharmacies of each investigational site. The supervising central data manager has access to the treatment allocation in Castor EDC, but is not involved in outcome assessment, data analysis, or any other steps that require blinding. Study team members entering data in Castor EDC have not been authorized to see treatment allocation in the web-based system, and therefore remain blinded when entering data. Pharmacy trial assistants and trial pharmacists (who do not take part in the study team) have authorization to see treatment allocation in Castor EDC, in order to allocate the right treatment to each patient. The trial pharmacies provide study medication using packaging and labeling ensuring blinding of study team members, healthcare providers, and participants.

#### Procedure for unblinding if needed {17b}

Study team members and healthcare providers do not have access to the treatment allocation code. The code may be broken only in circumstances when knowledge of the study medication is essential for treating the patient (e.g., SAE, SUSAR), if possible after consulting the principal investigator or sub-investigator. During office hours, unblinding of a participant can be done by contacting the pharmacy trial assistants. Outside office hours, the on-call pharmacist has to be contacted.

### Data collection and management

#### Plans for assessment and collection of outcomes {18a}

##### Study procedures and assessments

*Vital signs and physical examination*

According to standard care, systolic and diastolic blood pressure, heart rate, tympanic temperature, and saturation will be checked at baseline and four times daily during hospitalization. Physical examination will be conducted at baseline and daily thereafter during hospitalization.

*Mucositis scores*

Mouth and gut scores will be obtained daily during hospitalization, using the daily oral mucositis score (DMS) and modified daily gut score (M-DGS) respectively [28].

*Laboratory testing*

Blood for chemical and hematological analysis will be obtained at baseline and then thrice-weekly during hospitalization. Blood chemistry panel consists of sodium, potassium, magnesium, calcium, urea, creatinine, phosphate, albumin, alkaline phosphatase, direct bilirubin, total bilirubin, gamma-GT, ALAT, ASAT, LDH, and glucose. Hematological panel consists of hemoglobin (Hb), hematocrit (Ht), white blood cell count and differential, and thrombocytes.

*Blood and surveillance cultures*

Blood cultures will be drawn daily from day + 4 up until day + 12. This will be halted after the occurrence of fever or start of empirical antibiotics (ceftazidime). Additionally, blood cultures will be drawn in the occurrence of fever. Surveillance cultures of mucosal barriers are performed at admission and twice-weekly during hospitalization. Also, an oral and a rectal swab will be obtained at screening.

*Blood samples for translational studies*

During the study, blood samples for translational studies (citrulline, CRP, biomarkers) will be obtained. For this purpose, plasma will be obtained at baseline and then thrice-weekly during hospitalization.

*Microbiota*

For the evaluation of the microbiota, stool samples and oral swabs will be collected at day − 3 (day of admission) and twice-weekly during hospitalization.

*Use of supportive care*

The use and duration of use of total parenteral nutrition (TPN), opioid analgesics, and antibiotics will be recorded weekly.

*Questionnaires*

Quality of life and fatigue severity will be assessed at baseline and at day of discharge or day 30 of hospitalization, whichever comes first. Subsequently, these assessments will be done on day + 100 and + 1 year, for which the questionnaires are sent to patients (digital or to home address). For the assessment of quality of life, the validated EORTC-QLQ C30 questionnaire will be used [29]. For the assessment of fatigue severity, the validated FACIT-F Fatigue questionnaire will be used [30].

*Safety measurements:*

Apart from (S)AE reporting, non-hematological grade 3–4 side effects will be assessed and reported separately and graded according to the common toxicity criteria (CTCAE version 5.0). As important hematological safety outcome, period to neutrophil recovery will be recorded. Finally, serious infections and opportunistic infections will be recorded.

A tabulated overview of the study activities is given in Fig. 2.

#### Plans to promote participant retention and complete follow-up {18b}

There are no specific measures to promote participant retention. To promote complete follow-up (i.e., questionnaires at day + 100 and + 1 year), participants are sent a reminder if the questionnaires have not been returned yet after a few weeks.

In case of premature termination of the study, all subjects will undergo safety assessments as planned. All subjects who discontinued treatment will continue to be followed at standard intervals until 1 year after HSCT, or death, conforming to institutional protocol. Therefore, safety assessments of discontinued patients can be included in the analysis.

### Data management {19}

After inclusion, each participant will receive an individual, site-specific study code. Study data will be collected by site study team members in an electronic case report form (eCRF) designed for the study, using web-based data capture software (Castor EDC©) compliant with GCP requirements.

The central data manager monitors data entry of all sites and ensures that inaccurate or missing data are addressed as soon as possible after detection.

Risk-adjusted monitoring will be performed by personnel independent of the research team and will consist of checking informed consent forms, completeness of eCRFs, and source data verification for a random sample of patients.

Further details on the collection of data are described in a data management plan.

### Confidentiality {27}

All collected data will be kept strictly confidential and will be stored in accordance with the General Data Protection Regulation and Good Clinical Practice (GCP).

### Plans for collection, laboratory evaluation, and storage of biological specimens for genetic or molecular analysis in this trial/future use {33}

No genetic or molecular analysis will be done in this trial.

### Statistical methods

#### Statistical methods for primary and secondary outcomes {20a}

Primary efficacy analyses will be performed for both the intention-to-treat (ITT) and per-protocol (PP) populations. The ITT population will consist of all randomized subjects who have been administered at least one dose of study medication. The PP population will exclude all subjects in the ITT population who have taken any interfering concomitant medications or who have been given incorrect study medication (i.e., placebo for anakinra-group or anakinra for placebo-group) during the treatment phase (initial hospital admission) or who have missing data for one or more assessments of the primary outcome.

For the analysis of the primary study parameter, fever during neutropenia, the chi-square test will be used. For the analysis of repeated measurements (e.g., citrulline, mucositis score), mixed models will be used. Continuous variables will be summarized with sample size, mean, standard deviation, and range. Frequency counts and percentage of subjects within each category will be provided for categorical data. Safety will be evaluated by tabulations of AE/SAE and will be presented with descriptive statistics for each treatment group.

A detailed statistical analysis plan will be agreed to before the end of data entry and before the treatment code is broken.

#### Interim analyses {21b}

An interim analysis to evaluate safety will be performed after the first 40 subjects have completed study treatment, with at least 30 days follow-up. The study will be terminated prematurely if the interim analysis shows that treatment with the study medication is considered unsafe. The results of the interim analysis will be evaluated by the Data Monitoring Committee (DMC).

The stopping rules for prematurely ending the trial after interim analysis because of safety reasons will be the following:

- More infections due to opportunistic pathogens, e.g., Aspergillus spp., in the experimental group than the control group (difference between experimental and control group > 25%, only when incidence at least 2 in a group)
- More severe infections leading to transfer to the intensive care unit in the experimental group (difference between experimental and control group > 25%, only when incidence at least 2 in a group)
- More severe (CTCAE grade 3–4) non-hematological adverse events in laboratory parameters (e.g., renal impairment or increased liver enzymes) in the experimental group (difference between experimental and control group > 25%, only when incidence at least 4 in a group)
- Higher occurrence of primary graft failure or prolonged neutropenia in the experimental group (neutrophils have not been > 0.5 × 10^9^/l on one single day, assessed on day + 21, and counting from day 0).

#### Methods for additional analyses (e.g., subgroup analyses) {20b}

No subgroup or adjusted analyses have been planned.

#### Methods in analysis to handle protocol non-adherence and any statistical methods to handle missing data {20c}

Primary efficacy analyses will be performed for both the intention-to-treat (ITT) and per-protocol (PP) populations. The ITT population will consist of all randomized subjects who have been administered at least one dose of study medication. The PP population will exclude all subjects in the ITT population who have taken any interfering concomitant medications or who have been given incorrect study medication (i.e., placebo for the anakinra group or anakinra for the placebo group) during the treatment phase (initial hospital admission), or who have missing data for one or more assessments of the primary outcome.

#### Plans to give access to the full protocol, participant-level data, and statistical code {31c}

Anyone interested in documentation other than presented in this manuscript should contact the corresponding author.

### Oversight and monitoring

#### Composition of the coordinating center and trial steering committee {5d}

The coordinating center study team consists of the principal investigator, sub-investigators, statistician and research nurses, and a trial coordinator, data manager, and a central data manager of the Radboud Technology Center Clinical Studies (RTC CS). The coordinating trial steering committee (coordinating TSC), consisting of the principal investigator and three sub-investigators from the Radboudumc, oversees the project and meets every 3 months. The general TSC consists of the coordinating TSC and the principal investigators of the participating centers.

#### Composition of the data monitoring committee, its role and reporting structure {21a}

The data monitoring committee (DMC) will oversee trial safety and includes independent members with clinical and methodological expertise. The DMC consists of a hematologist, a medical microbiologist, and a statistician and reports to the TSC. The DMC will advise whether it is safe to continue the study after the interim analysis. Also, the DMC will evaluate every SAE in the first 40 patients. If no severe complications occur, the frequency of SAE evaluation by the DSMB can be reduced. Details on the DMC are given in a separate document: DSMB charter.

#### Adverse event reporting and harms {22}

##### Adverse events

Adverse events (AEs) reported spontaneously by the patient or observed by the investigator or his staff will be recorded. Pre-existing relevant conditions will be collected on the medical history CRF. Adverse events will be recorded from day of admission (day − 3) until 30 days following the last dose of the investigational treatment. AEs will be scored according to the NCI Common Terminology Criteria for Adverse Events (CTCAE), version 5.0.

All adverse events have to be recorded, with the exception of the following: a pre-existing condition that does not increase in severity, AEs of CTCAE grades 1 and 2, and abnormal laboratory values that have been recorded as being not clinically significant by the investigator in the source documents.

##### Serious adverse events (SAE)

In the event of an SAE, the investigator may immediately stop treatment if it is considered in the best interest of the patient. In case a serious adverse event related to the study medication occurs, the patient may be withdrawn, unless doing so would harm the patient in the opinion of the investigator.

The investigator will report all SAEs to the sponsor, without undue delay after obtaining knowledge of the events, except for the following SAEs:

- Elective hospitalization for pre-existing conditions that have not been exacerbated by trial treatment
- Hospitalization for protocol therapy administration. Hospitalization or prolonged hospitalization for a complication of therapy administration will be reported as a SAE
- Hospitalization (for a procedure) that was planned prior to study participation (i.e., prior to registration of randomization). This should be recorded in the source documents. Prolonged hospitalization for a complication of such procedures remains a reportable SAE
- Hospitalization for diagnostic investigations (e.g., scans, endoscopy, sampling for laboratory tests, bone marrow sampling) that are not related to an adverse event. Hospitalization or prolonged hospitalization for a complication of such procedures remains a reportable SAE
- Hospitalization for blood or blood product transfusion
- Medical or surgical procedure (e.g., endoscopy, appendectomy); the condition that leads to the procedure is an (S)AE
- Hospitalization or prolonged hospitalization for technical, practical, or social reasons, in absence of AEs
- Clinical events related to the primary cancer progression are not to be reported as SAEs, even if they meet any of the seriousness criteria from the standard SAE definition, unless the event is more severe than expected and therefore the investigator considers that their clinical significance deserves reporting
- Relapse/progression of the disease under study; death or complications as a result of disease progression remain reportable SAEs.

SAEs must be reported to the sponsor within 24 h after the event was known to the investigator, in a report containing the following information on NCI-CTCAE grade, start date, seriousness criterium, causality with study treatment, action taken with regard to study treatment, and associated treatment. After sending the SAE form, it should also be recorded on the AE form of the eCRF.

The sponsor will report the SAEs to the relevant regulatory bodies (the METC that approved the protocol), within 7 days of first knowledge for SAEs that result in death or are life threatening followed by a period of maximum of 8 days to complete the initial preliminary report. All other SAEs will be reported within a period of maximum 15 days after the sponsor has first knowledge of the serious adverse events.

SAEs will be reported from day of admission (day − 3) until 30 days following the last dose of the investigational treatment. SAEs occurring after 30 days should also be reported if considered at least possibly related to the investigational medicinal product by the investigator.

##### Suspected unexpected serious adverse reactions (SUSARs)

Adverse reactions are all untoward and unintended responses to an investigational product related to any dose administered.

Unexpected adverse reactions are SUSARs if the following three conditions are met:

1. The event must be serious
2. There must be a certain degree of probability that the event is a harmful and an undesirable reaction to the medicinal product under investigation, regardless of the administered dose;
3. The adverse reaction must be unexpected, that is to say, the nature and severity of the adverse reaction are not in agreement with the product information as recorded in:
  Summary of product characteristics (SPC) for an authorized medicinal product;
  Investigator’s Brochure for an unauthorized medicinal product.

The coordinating PI of the study reviews every reported SAE and decides if it is a SUSAR or not. The sponsor will report expedited the following SUSARs to the METC:

- SUSARs that have arisen in the clinical trial performed according to this protocol that was assessed by the METC;
- SUSARs that have arisen in other clinical trials with the same medicinal product and that could have consequences for the safety of the subjects involved in the clinical trial that was assessed by the METC.
- The remaining SUSARs (from other clinical trials with the same product) that have no consequences for the safety of the subjects will be reported to the METC, if they are made available by the pharmaceutical company. This will be reported as a line-listing together with the safety report.

The expedited reporting will occur not later than 15 days after the sponsor has first knowledge of the adverse reactions. For fatal or life-threatening cases, the term will be maximal 7 days for a preliminary report with another 8 days for completion of the report.

##### Annual safety report

In addition to the expedited reporting of SUSARs, the sponsor will submit, once a year throughout the clinical trial, a safety report to the accredited METC and competent authority according to the guidelines of the METC.

##### Follow-up of adverse events

All AEs will be followed until they have abated, or until a stable situation has been reached. Depending on the event, follow-up may require additional tests or medical procedures as indicated, and/or referral to the general physician or a medical specialist.

Follow-up information on SAEs should be reported monthly until recovery or until a stable situation has been reached. The final outcome of the SAE should be reported on a final SAE report.

### Frequency and plans for auditing trial conduct {23}

The trial office monitors aspects of the study on an ongoing basis as described, for example in the data management paragraph. The trial, including all participating centers, is monitored and audited by a certified monitor from the RTC CS Nijmegen, which is independent from the department of hematology and study team. Details on monitoring are given in a separate document: monitoring plan.

### Plans for communicating important protocol amendments to relevant parties (e.g., trial participants, ethical committees) {25}

All substantial amendments will be discussed within the TSC and all principal investigators and will be notified to the METC, competent authority, DMC, and relevant trial registries and journals. Also, trial participants will be informed on important protocol modifications if personally relevant for them (e.g., change of study procedures during study participation).

### Dissemination plans {31a}

Research results will be disseminated to all stakeholders, including clinicians, scientists, and patients. This will be done through publications in peer-reviewed journals, presentations at (inter)national conferences and symposia, and the participation in (inter)national expert networks such as HOVON (Hemato-Oncology for Adults in the Netherlands; Dutch: Hemato-Oncologie voor Volwassenen Nederland) and MASCC (Multinational Association for Supportive Care in Cancer). Patients will be informed via Hematon, the Dutch patient organization for patients with hemato-oncological disorders. Furthermore, project information and results will be available for patients via websites (www.kanker.nl) and through public information presented by the funding body DCS.

## Discussion

Fever during neutropenia, infections, and mucositis remain frequently occurring and important complications in the treatment of patients with intensive chemotherapy and HSCT. Nevertheless, there is an unmet need for the prevention and management of these inflammatory complications. Animal studies have shown that inhibition of IL-1 may be an effective strategy in reducing mucositis and related inflammatory complications. This study will be the first randomized placebo-controlled trial to evaluate the effect of inhibition of IL-1 using anakinra in the management of fever during neutropenia and mucositis, specifically in patients with multiple myeloma, treated with high-dose melphalan and HSCT.

If proven effective in reducing the incidence of FN and related inflammatory complications, it is expected that, consequently, the use of antimicrobial agents can be reduced. This is particularly important considering the threat posed by increasing antimicrobial resistance and the negative impact of intestinal dysbiosis on survival of HSCT recipients.

Several comments can be made regarding theoretical risks of the use of IL-1 inhibition in this patient population. However, we believe that these can be sufficiently refuted by evidence available from literature.

The most serious risk associated with anakinra is an increase in the incidence of infections (reported incidence approximately 2%). These usually consist of bacterial infections, and most infections concerned pneumonia and cellulitis. IL-1β plays an important role in the host defense against bacteria and fungi. Also, it provides partial protection against infections with intracellular bacteria, such as Salmonella, Listeria, and *Mycobacterium tuberculosis* [31–34]. However, virtually no increase in the rate of opportunistic infections has been observed in clinical studies and clinical practice [35, 36].

Furthermore, neutropenia is observed as a side effect of anakinra in approximately 2% of patients [37]. Even so, patients in this study will inevitably develop neutropenia because of their treatment with intensive chemotherapy which requires a HSCT. Concerns might therefore arise regarding an impact of anakinra on stem cell engraftment. However, in previous studies in HSCT recipients, anakinra at high doses (up to 3200 mg/day) did not prolong neutropenia [38] and time to neutrophil recovery was similar in anakinra-treated and placebo-treated groups [39].

Finally, concerns may arise regarding the effect of IL-1 inhibition on the effect of anti-myeloma therapy.

IL-1 is involved in the pathogenesis of multiple myeloma: it stimulates the development and progression of multiple myeloma [40–44]. Anakinra has been studied in patients with premalignant stages of multiple myeloma [monoclonal gammopathy of undetermined significance (MGUS) and smoldering multiple myeloma (SMM)], in which it was shown to be both safe and effective [45, 46]. Therefore, treatment with anakinra is not expected to have a negative effect on anti-myeloma therapy.

In conclusion, this study will show whether treatment with IL-1 is effective in the management of fever during neutropenia and mucositis in this vulnerable patient population. Furthermore, this study will give more insight in the pathophysiology of mucosal barrier injury. If treatment proves to be effective, this well be an important step forward in the management of patients at risk for these disabling complications.

## Trial status

Protocol version 1.1, 29-05-2019

Radboudumc: recruiting (start: 23-09-2019)

Amsterdam UMC and UMCG: not yet recruiting (study in preparation)

Approximate date when recruitment will be completed: 23-09-2022

## Abbreviations

AE: Adverse event
ALAT: Alanine aminotransferase
ANC: Absolute neutrophil count
ARDS: Acute respiratory distress syndrome
ASAT: Aspartate aminotransferase
CAPS: Cryopyrin-associated periodic syndromes
CPE: Carbapenemase-producing Enterobacteriaceae
CRP: C-reactive protein
CVC: Central venous catheter
DCS: Dutch Cancer Society
DMC: Data monitoring committee
DMS: Daily oral mucositis score
eCRF: Electronic case report form
EDC: Electronic Data Capture
EORTC-QLQ: European Organization for Research and Treatment of Cancer – Quality of Life Questionnaire
EudraCT: European drug regulatory affairs Clinical Trials
FACIT: Functional Assessment of Chronic Illness Therapy
FN: Fever during neutropenia
GCP: Good Clinical Practice
GI: Gastrointestinal
HDM: High-dose melphalan
HOVON: Hemato-Oncology for Adults in the Netherlands (Dutch: Hemato-Oncologie voor Volwassenen Nederland)
HSCT: Hematopoietic Stem Cell Transplantation
IL-1: Interleukin-1
IL-1RA: Interleukin-1 receptor antagonist
IL-1RI: Interleukin-1 receptor type I
ITT: Intention to treat
LDH: Lactate dehydrogenase
MAMP: Microbe-associated molecular pattern
MASCC: Multinational Association for Supportive Care in Cancer
MBI: Mucosal barrier injury
M-DG: Modified Daily Gut Score
METC: Medical research ethics committee (MREC) [Dutch: Medisch Ethische Toetsing Commissie (METC)]
MGUS: Monoclonal gammopathy of undetermined significance
NaCl: Sodium chloride
NSAIDs: Non-steroidal anti-inflammatory drugs
MRSA: Methicillin-resistant *Staphylococcus aureus*
PP: Per protocol
RA: Rheumatoid arthritis
RTC CS: Radboud Technology Center Clinical Studies
SC: Stem cell
SAE: Serious adverse event
SMM: Smoldering multiple myeloma
SPC: Summary of product characteristics
SUSAR: Suspected unexpected serious adverse event
TPN: Total parenteral nutrition
TSC: Trial steering committee
ULN: Upper limit of normal
VRE: Vancomycin-resistant enterococci
